# Lateral Inhibition in Accumulative Computation and Fuzzy Sets for Human Fall Pattern Recognition in Colour and Infrared Imagery

**DOI:** 10.1155/2013/935026

**Published:** 2013-10-31

**Authors:** Antonio Fernández-Caballero, Marina V. Sokolova, Juan Serrano-Cuerda

**Affiliations:** ^1^Instituto de Investigación en Informática de Albacete (I3A), 02071 Albacete, Spain; ^2^Departamento de Sistemas Informáticos, Universidad de Castilla-La Mancha, 02071 Albacete, Spain; ^3^Southwest State University, 94, 50 let Oktyabrya, Kursk 305040, Russia

## Abstract

Fall detection is an emergent problem in pattern recognition. In this paper, a novel approach which enables to identify a type of a fall and reconstruct its characteristics is presented. The features detected include the position previous to a fall, the direction and velocity of a fall, and the postfall inactivity. Video sequences containing a possible fall are analysed image by image using the lateral inhibition in accumulative computation method. With this aim, the region of interest of human figures is examined in each image, and geometrical and kinematic characteristics for the sequence are calculated. The approach is valid in colour and in infrared video.

## 1. Introduction

Human posture recognition is one of the core problems in pattern recognition for computer vision, as it has become an essential basic component of a greater part of application problems such as ambient intelligence, surveillance, action recognition, human-computer interaction, elderly health care, and others [1–3]. One of the particular tasks for human posture recognition is fall detection. Correct and rapid recognition of a fall carries an important meaning for applications in assisted living, for example, of elderly people [4]. As many falls occur during the night, when low illumination or its absence is one of the factors that facilitate falls, infrared video cameras are used.

There are various methods of fall detection, which include usage of wearing devices, sensor-based approaches (smart rooms, sensible floors), and methods of visual data processing [5]. The first group of methods suppose that a person wears a miniaturized device, which enables recollection of parameters and launches an alarm in case a fall is detected. A wide type of accelerometers or movement/vibration sensors belongs to this group [6]. Although the usage of wearable devices has many positive characteristics, some authors point out the shortcomings of this approach such as reluctance and negligence to wear sensors, and the tendency of wearing devices to produce false alarms [7]. The second group includes various solutions to detect a fallen person, which imply sensors placed in the usual place surrounding the person. It includes solutions based on floor vibration and acoustic sensing [8], through the installation of sensitive floor tiles [9]. As these sensors are fixed or integrated into a given environment, they cannot be moved easily when a person changes position. In general, acoustic and vibration sensors are expensive and fragile as well as may request special conditions for correct functionality. The visual data processing methods are free from the limitations of the previously described groups of methods. This group combines various solutions, such as head motion analysis, shape motion analysis, and inactivity detection [10].

In this paper we present an approach for human fall recognition which is based on fuzzy patterns for various fall types. We introduce a method for posture recognition and fall detection given an output of infrared video sequence. The presented method is based on the lateral inhibition in accumulative computation approach for colour and infrared video segmentation and on fuzzy fall detection mechanism, which creates a fuzzy model of fall patterns as function of geometrical, temporal, and kinematic parameters of a video sequence [11–13]. Indeed, lateral inhibition in accumulative computation (LIAC) has proved to be an efficient method for moving object segmentation in grey-level video sequences [14–16] and has been implemented in real-time [17]. Due to its versatility, the LIAC method has been applied successfully to dynamic visual attention [18] in surveillance applications [19] in order to monitor human activities. Also some works have provided enhancements through the inclusion of genetic algorithms [20] and stereoscopy [21, 22].

The rest of the paper is structured as described next.  Section 2.1 introduces the LIAC method in the motion detection task in colour and infrared video.  Section 2.2 explains the fuzzy fall detection module. Then, Section 3 introduces a couple of examples which show the effectiveness of the approach. Lastly, in Section 4 the most important conclusions are offered.

## 2. Materials and Methods

### 2.1. Lateral Inhibition in Accumulative Computation

**Figure 1:**
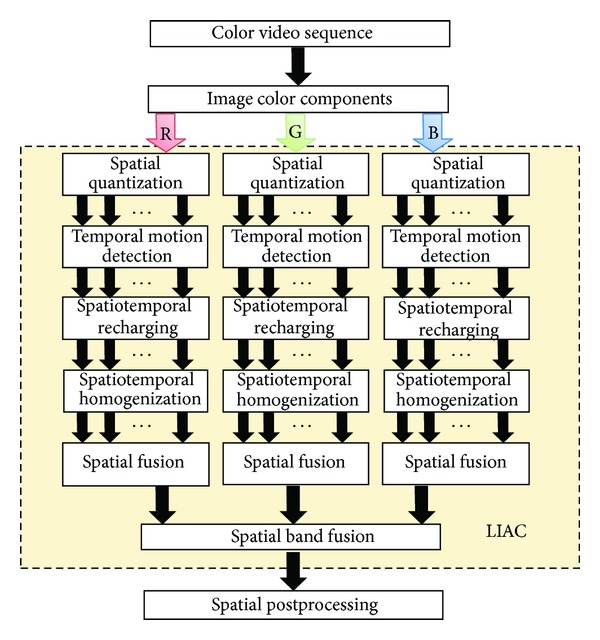
LIAC architecture for colour video sequences (for RGB in this case).

The problem we are stating by means of lateral inhibition in accumulative computation is the discrimination of moving objects capable of holding our attention in a scene. Motion allows gradually obtaining all moving objects' shapes through a mechanism called accumulative computation. Then, the algorithm fuses spots obtained by means of neurally-inspired lateral inhibition (LI) and thresholding. The complete LIAC architecture is shown in Figure 1, where the reader may have a first contact with the modules of the method. 

The adaptation of the LIAC algorithm to colour video requires expanding from one unique grey-level component to three colour components of the colour space used. For infrared video, there is no need for adaptation. Next, each one of the modules is described in detail. Also, the influence of the most important parameters of the LIAC algorithm are briefly explained. A more detailed explanation of the parameters is available in a previous work [23].

#### 2.1.1. Spatial Quantization

The module covers the need to segment the input image into a preestablished group of bands (*N*) (vector quantization [24]). A high value of *N* usually enables us to better discriminate the whole shapes of the moving nonrigid objects. Nevertheless, a too high value of this parameter may include some image background into the shapes. This may even lead to fuse more than one different shape into one single silhouette.

#### 2.1.2. Temporal Motion Detection

Now, a charge or discharge due to motion detection is performed. This module has been designed to obtain the accumulated charge on a quantization basis in 3 layers (colour components) or 1 layer (infrared component), and each one of them will memorize the value of the accumulative computation present at time scale *t* for each pixel.

 At each pixel (*x*, *y*) we are in front of three possibilities:

1. the charge value at pixel (*x*, *y*) is discharged down to the minimum allowed charge value when no motion information may be detected at a given band. No motion information is available as pixel (*x*, *y*) does not correspond to that band;
2. the charge value at pixel (*x*, *y*) is saturated to the maximum charge value when motion is detected at *t*. Motion is detected as image pixel now belongs to this band at time instant *t*, and it did not correspond to the band at the previous instant *t* − Δ*t*, or vice versa;
3. the charge value at pixel (*x*, *y*) is decremented by a given value when motion goes on being detected in consecutive intervals *t* and *t* − Δ*t*. Of course, the permanence value cannot get off a minimum value. Notice that the discharge of a pixel by a quantity is the way to stop maintaining attention to a pixel of the image that did capture our interest in the past. As it will be seen later on, if a pixel is not directly or indirectly bound by means of lateral inhibition mechanisms to a maximally charged pixel, it goes down to the total discharge with time. The influence of the discharge value is as follows. Different values of the discharge value due to motion detection offer different trails of the movement in the consecutive output images. When lowering the discharge value, more information of the history of the movement is obtained through the offered trail.

#### 2.1.3. Spatio-Temporal Recharging

Lateral inhibition is thought here to reactivate the accumulated charge with an extra recharge of those pixels which are partially loaded and directly or indirectly connected to maximally charged pixels. Spatio-temporal recharging occurs in steps after t and before the next frame.

In order to explain the notion of this step, we will say that the activation toward the lateral modular structures (up, down, right, and left) is based on the following basic ideas:

1. all modular structures with maximum accumulated charge value output the charge toward the neighbours;
2. all modular structures with a nonsaturated charge value allow passing this information through them if activated by some neighbour (they behave as transparent structures to the charge passing);
3. the modular structures with minimum permanence value stop the passing of the charge information toward the neighbours (they behave as opaque structures). Therefore, we are in front of an explosion of lateral activation which begins at the structures with accumulated charge and spreads lineally toward all directions, until a structure appears in the pathway with a complete discharge. One important issue is that the recharge at each pixel takes place at most once.

Notice that the recharge has a secondary effect, recovering part of the history of motion. The accumulated charge of each pixel will be offered to the following module as output.

#### 2.1.4. Spatio-Temporal Homogenization

In this module the charge is distributed among all the connected neighbours holding a minimum charge, once again by means of lateral inhibition mechanisms. The explanation of this data clustering-based method is as follows. Starting from the values of the accumulated charge values in each pixel on a band basis, we will see how it is possible to obtain all the parts of a moving object. A part of an object is just the union of pixels that are together and in a same band.

The charge is homogenized among all the pixels that pertain to the same band and that are directly or indirectly united to each other. This way, a double objective will be obtained: (1) diluting the charge due to the false image background motion along the other pixels of the background; so, there should be no presence of the motion characteristic of the background, but we will rather keep motion of the objects present in the scene and (2) obtaining a parameter common to all the pixels of the part of the object in a surrounding window with a same band.

#### 2.1.5. Spatial Fusion

During this step, we take the maximum value of all outputs of all bands to show the detected blobs associated with a moving object as obtained for each colour component.

#### 2.1.6. Spatial Band Fusion

In the RGB colour space the final output segmentation result is obtained as a logical AND of the three partial outputs. 

#### 2.1.7. Spatial Postprocessing

This module performs a binarisation with a given threshold. Values over threshold are set to max (255) and below threshold are set to min (0). Once the image is binarised, some morphologic operations leading to eliminate image noise are performed. Firstly, erosion is performed in order to eliminate isolated and small spots. And, secondly, a dilation operation is computed to enhance the remaining spots. Finally, spots are filtered based on their features, such as height, width, and compactness. For this purpose, minimum and maximum values are established.

### 2.2. Fall Pattern Recognition

The proposed fuzzy model detects a fall and a fall pattern from the following list: falls from a “standing” position, falls from a “sitting” position, and falls from a “lying” position; and indicates the direction (lateral right, lateral left, backward, and forward) and velocity of a fall. The system does not require any offline training, it calibrates a person and then launches the fall detection mode, analysing video frames every 1 to 3 seconds (“fall time”) [25]. Once a fall is detected and recognized, the system changes to inactivity monitoring mode, and, if a person does not stand up during the following 30 seconds, an alarm signal is generated. Below are the following fall patterns that are recognized so far.

1. Static and dynamic falls.
2. Falls from the most usual human positions, namely, “standing,” “sitting,” and “lying.”
3. Forward, backward, and lateral falls (to the left and to the right).
4. False falls which correspond to other human positions such as “kneeling,” “crouching,” and “squatting.”

#### 2.2.1. Determination of Fall Indicators

A fall is determined as dropping or coming down freely under the influence of gravity and can be described by several parameters, which describe spatial and temporal positions of the person in a sequence of video images. For the *n* consecutive ROIs corresponding to a same human, we consider the following spatial parameters.

- “Width to Height ratio” parameter is calculated for each ROI. In the case of a standing person it is usually less than a “0”. As a person started to loose erect position, falling down, or bending, this parameter increases. Our experiments have shown that its value for a lying person belongs to interval ∈[1.5 ⋯ 7.0].
- “Height change” parameter is calculated for the first and the last images in a sequence of images stored for a given fall time. It is a relation of the height of the ROI of the last image to the ROI of the first image.
- “Fall direction” parameter is calculated for the first and the last images in a sequence of images stored for a given fall time. It returns a sign of the difference between the upper left corner of the ROI from the first image and the corresponding coordinate of the ROI from the last image. In case of a negative value, the fall direction is “to the left,” and in case of a positive value, it is “to the right;”
- “Position change” parameter indicates, first, if the upper left corner of the ROI of the last image of the sequence is higher than the corresponding ROI of the first image. “Position change” is equal to “1” in case these statements are true and to “0” otherwise. This parameter was specially introduced in order to facilitate detection of the fall from a bed, or, in other words, falls from a “lying” position.

We also consider the following kinetic parameters, which are calculated between the ROIs of the first and the last images in a sequence of images stored for a fall time.

- “Horizontal velocity of a fall” describes a ratio between the real horizontal velocity (equal to the factual height change between the first and the last images, ROIs) and the maximum possible horizontal velocity (equal to the height of the ROI from the first image).
- “Vertical velocity of a fall” describes a ratio between the real vertical velocity (equal to the factual width change between the first and the last images ROIs) and the maximum possible vertical velocity (equal to the width of the ROI from the first image).

#### 2.2.2. Fuzzy System Design

**Figure 2:**
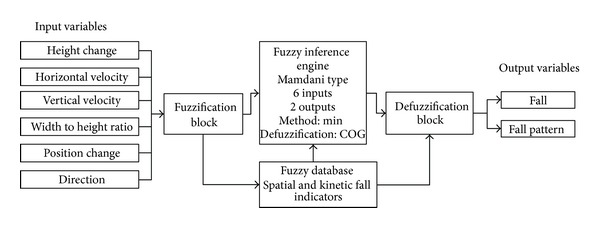
Fuzzy inference system for fall patterns recognition.

After the previous parameters have been calculated, fuzzy logic [26] is used in the proposed fall detection subsystem to determine the ranges for the fall indicators and to classify one of the possible fall patterns. Fuzzy logic eases the problem of fall detection, on the one hand, and facilitates recognition of fall patterns, on the other hand.  Figure 2 gives a schematic view on our fuzzy inference system, which executes as follows.

 Initially, input crisp variables are transformed into fuzzy sets within the “Fuzzification block.” Next, the “Fuzzy inference engine” simulates the reasoning process by making fuzzy inference on the inputs and fuzzy “IF-THEN” rules, which are stored within the “Knowledge base,” which includes fuzzy rules and cases from “Fuzzy database.” Lastly, the fuzzy set obtains the crisp values corresponding to the output variables.

We used the fuzzy logic library Fuzzylite (see http://code.google.com/p/fuzzy-lite/), which provides a set of classes and methods for fuzzy interference system creation and manipulation. The fuzzy system is coded in agreement with the Fuzzy Control Language, which is a standard for Fuzzy Control Programming published by the International Electrotechnical Commission (IEC) (see http://www.ansi.org/). Linguistic variables describe spatial and kinetic properties of ROIs, which correspond to humans. Our fuzzy model for fall detection includes six input linguistic variables described previously, that is, “HeightChange,” “HorizontalVelocity,” “VerticalVelocity,” “WidthToHeightRatio,” “PositionChange,” and “FallDirection,” and two output linguistic variables called “Fall” and “FallPattern.”

The fuzzy sets for the input linguistic variables “HeightChange,” “HorizontalVelocity,” “VerticalVelocity,” and “WidthToHeightRatio” include three fuzzy terms, namely, LOW, MEDIUM, and HIGH. 

Variable “HeightChange” is calculated as the relation of the initial height to the final height of the ROI. As stated in [27], the width of a human's body is around 25% of his/her height. Taking into account different human builds, we have experimentally decided to set up a value of 30% of an ROI's height as the higher boundary for the HIGH term of linguistic variable “HeightChange.” Variables “VerticalVelocity” and “HorizontalVelocity” are calculated as the relation of the real velocity to the maximum possible velocity. Thus, in case of a very quick fall with velocity equal or close to the maximum, horizontal, and vertical velocity, *≈*1.25 is gotten. On the contrary, the change of velocity is minimal when velocity is *≈*0.0. The terms for these linguistic variables have the following ranges: LOW ∈[0 ⋯ 0.35], MEDIUM ∈[0.25 ⋯ 0.65], and HIGH ∈[0.6 ⋯ 1.25]. The “WidthToHeightRatio” variable ranges from 0.0 to 6.0. Since an ROI corresponding to a person in a strengthened position is longer in height than in width, the term LOW ∈[0 ⋯ 0.8] and the term MEDIUM ∈[0.7 ⋯ 2.5] stand for an situation where an ROI belongs to a fallen person, or, alternatively, to a sitting or prone person. Finally, the term HIGH ∈[2.1 ⋯ 6.0] describes an ROI of a lying person. The “PositionChange” and “FallDirection” variables are represented with crisp sets “0” and “1” for each variable.

The output linguistic variable “Fall” is represented with fuzzy terms “NO” ∈[0 ⋯ 45.0] and “YES” ∈[35.0 ⋯ 100]. The “FallPattern” linguistic variable includes the set of seven crisp values which correspond to the following fall patterns.

1. Falling backward or forward from a “standing” position.
2. Falling to the right from a “standing” position.
3. Falling to the left from a “standing” position.
4. Falling backward or forward from a “sitting” position.
5. Falling to the right from a “sitting” position.
6. Falling to the left from a “sitting” position.
7. Falling backward or forward from a “lying” position.

The fuzzy system reasons about the state of person and the first output variable, “Fall,” contain the resulting response of the system with two membership values corresponding to the confidence of a fall or bring upright. The second output variable, “FallPattern,” generates a crisp value ranged from 1 to 7, which indicates a pattern.

## 3. Results and Discussion

The following experiments were set up to validate the proposed approach. The experiments were carried out on an Intel Core i7 computer with 3 GB of RAM under Windows XP operating system. The infrared video sequences were recorded with a FLIR A320 camera at a resolution of 720 × 480 pixels, whereas, colour sequences were captured by a Sony FCB-EX780BP colour camera. Nine series of a single person for the infrared camera and five series of a single person for the colour camera were used to test if the algorithms presented achieve the desired results.

The results of possible fall types are introduced in the following subsections and their corresponding tables. The results include fall situations detected both from infrared and colour camera. These are exactly the falls which were simulated: static and dynamic falls from “standing” and “sitting” positions, fall from “lying” position, and a false fall. The capture of the videos was done with an interval of 200 milliseconds; the fall time was set to 1.2 seconds. Thus, every six consecutive frames were tested for fall detection.

**Figure 3:**
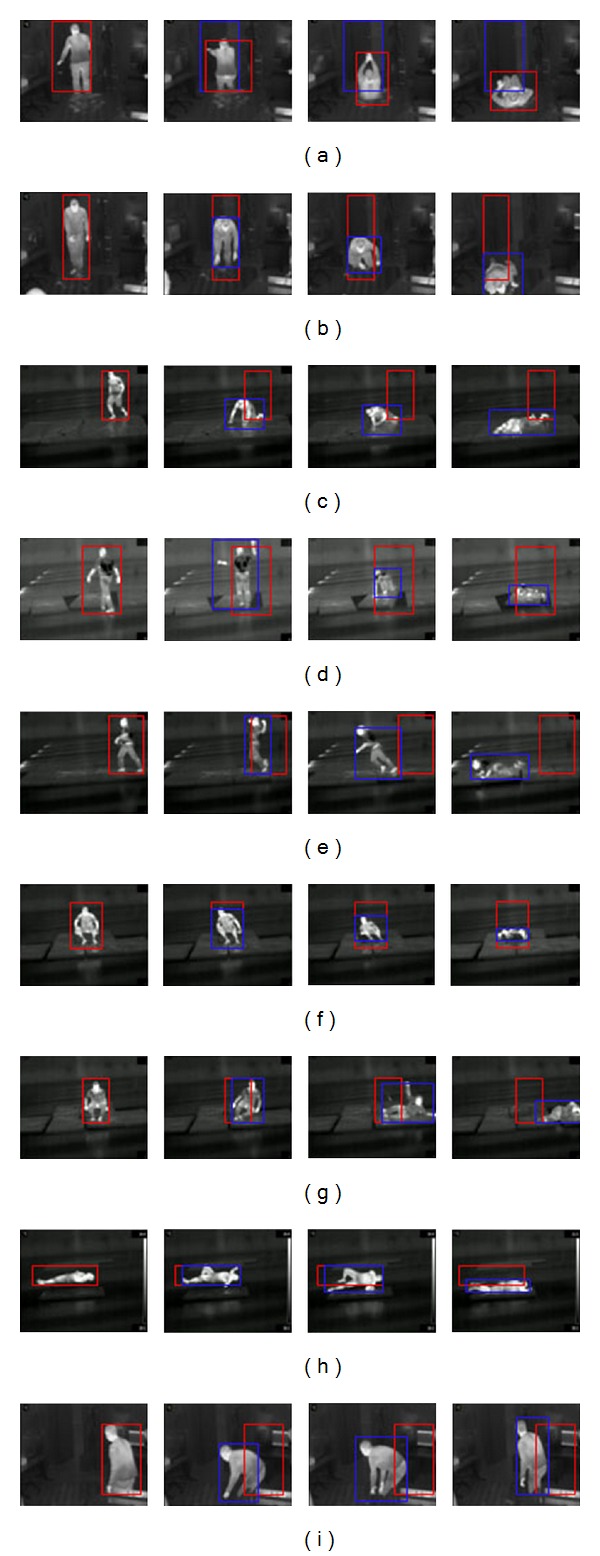
True and false falls recorded with an infrared camera. Static falls from a “standing” position: (a) falling backward, (b) falling forward and (c) falling to the left. Dynamic falls from a “standing” position: (d) falling forward and (e) falling to the left. Falls from a “sitting” position: (f) falling backward and (g) falling to the right. (h) Falling from the “lying” position. (i) False fall: “kneeling.”

**Figure 4:**
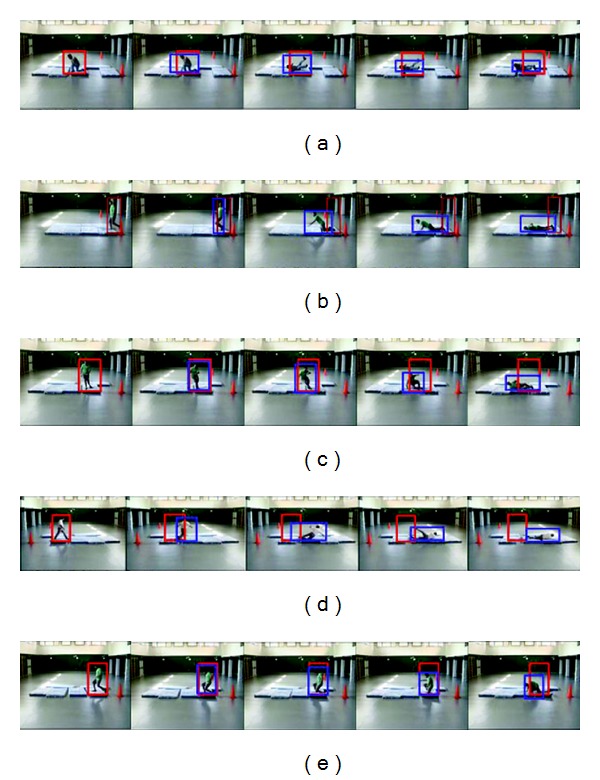
True and false falls recorded with a colour camera. Static fall from a “pruning” position: (a) falling backward. Static falls from a “standing” position: (b) falling forward and (c) falling to the left. Dynamic falls from a “standing” position: (d) falling to the right and (e) falling to the left.

The columns of result tables contain values of the input and the output variables of the fuzzy system for each time period of a possible fall. The column “Fall” shows the response of the model in the form of the fuzzy value, and the “FallPattern” column contains the number of a recognized pattern. Figures 3 and 4 show images with moments of falls (in infrared and colour video, resp.) which were used in the experiment. There are red and blue bounding boxes on the images. The red bounding box sets borders of the blob at the beginning of the fall time and the blue bounding box retains the borders corresponded to the first blob. Thus, we calculate fall indicators using the red and the blue boxes.

### 3.1. Detection of Static and Dynamic Falls from a “Standing” Position

Static falls include falling from the standing position and dynamic falls occur when a person moves before fall. Falls from the standing position are characterized with high horizontal velocity and height change. Width to height ratio changes significantly, too. We used a dynamic fall from a walking position to test the algorithms. This type of fall has the same characteristics as a static fall from the standing position, though velocity values are higher than in the case of static falls. 

Figures 3(a), 3(b), and 3(c) show fall moments for three static falls: for a person falling backward, falling forward, and falling to the left. The fall was detected with a highest value 67.5, which corresponds to the “YES” fuzzy term of the “Fall” linguistic variable. “FallPattern” is recognized correctly as “1,” which corresponds to “Falling backward or forward from the “standing” position.” The second fall is recognized as “1”—“Falling backward or forward from the “standing” position,” although the fuzzy response for the variable “Fall” changed from 65.44 to 35.81 and finally, has stabilized in “YES” fuzzy term (67.5). The fall to the left was also detected and recognized correctly (as marked “3” for the “FallPattern” variable).  Figure 3(d) shows frames with a moment of fall for a walking person who is falling forward. The fall was detected correctly with the “Medium” values for “velocity” and “width to height ratio” parameters as given in Table 1. Dynamic fall to the left is correctly classified as “3”—“Falling to the left from the “standing” position” (see Figure 3(e)).

The falls shown in Figures 4(b) and 4(c) were correctly recognized as “1”—“Falling backward or forward from the “standing” position,” having given a fuzzy response “YES” (67.5), and as “3”—“Falling to the left from a “standing” position.” Dynamic falls given in Figures 4(d) and 4(e) were also correctly detected and related to the patterns “1” and “2.”

### 3.2. Fall Detection from a “Sitting” Position

Falls from a sitting position are given in Figures 3(f) and 3(g) in infrared. The first fall is characterized with rapid increase of horizontal and vertical velocities. The fall to the right was classified correctly as having value “1” for “Fall direction” variable (see Table 2). Fall characteristics for the case of a pruning position are similar to ones of the sitting position, as acceleration of velocity is more important here than height change. A fall from a pruning position is given in Figure 4(e) and the results are offered in Table 2.

### 3.3. Fall Detection from a “Lying” Position

This fall (see Figure 3(h)) was recognized as “7”—“Falling to the left from the “lying” position,” because it has value “1” for “Position change” variable as well as “HIGH” values for the “WidthToHeightRatio” input variable (see Table 3).

### 3.4. Detection of False Falls

False falls are represented with kneeling. In fact, a false fall may be detected as a fall which lasts more than a fall time as well as having small values for fall detection parameters given in Table 3. As a rule, in case of a false fall, linguistic variable “width to height ratio” may have “LOW” or “MEDIUM” values, and linguistic variable “velocity change” is rather “LOW.” This fall was not detected (the variable “Fall” changed from 32.5 to 22.45, which corresponds to the “NO” term) neither classified.  Figure 3(i) shows a moment of a false fall. In compliance with the results given in Table 3, a fall is not detected in this case. Thus, the algorithms proposed were able to detect falls from false falls.

## 4. Conclusions

The elderly fall is an emergent problem, which needs fast and effective solutions. In this paper we have presented a system, which includes a human detection algorithm based in lateral inhibition in accumulative computation, and a fuzzy-based fall detection and inactivity monitoring model for colour and infrared video. The data supplied for the human detection algorithms are used later by the fuzzy model to detect if really a fall has occurred. The geometrical characteristics of the blob corresponding to the detected person, and the velocity of the change of its bounding box serve as fall indicators. Additionally, the fuzzy model enables to avoid certain limitations in parameter evaluation and to make smoother and more flexible decisions, on the other hand.

The proposed algorithms were incorporated into a fall detection system for the elderly, and then tested for a wide number of static and dynamic falls, including a test for false falls. The system may perform both efficiently at day time and night time, because of the mutual benefits of using colour and infrared cameras. The experimental results which were carried out for static and dynamic falls have demonstrated that the algorithms proposed are able to detect these situations and to distinguish real falls from false ones.

## Figures and Tables

**Table 1:** Results of fall detection and fall pattern recognition from a “standing” position in infrared and colour.

	Width to height ratio	Height change	Horizontal velocity	Vertical velocity	Fall direction	Position change	Fall	Fall pattern
Static fall Standing position Falling backward, Figure 3(a)	1.14 1.44 1.75	1.31 1.25 1.40	0.055 0.071 0.087	0.583 0.571 0.610	0 0 0	0 1 0	67.5 67.5 67.5	1

Static fall Standing position Falling forward, Figure 3(b)	1.19 1.32 1.43 1.53 1.68	1.02 1.21 1.12 1.12 1.07	0.215 0.000 0.000 0.037 0.560	0.914 0.177 0.322 0.560 0.560	0 1 0 0 0	0 0 1 1 0	65.4 38.6 39.5 35.8 67.5	1

Static fall Standing position Falling to the left, Figure 3(c)	1.50 2.54 2.95 3.27 2.93 3.31 3.38 4.53	1.73 1.16 1.18 1.08 1.34 1.46 1.62 1.00	0.024 0.544 0.550 0.847 0.544 0.544 0.544 0.000	0.332 0.072 0.084 0.096 0.151 0.163 0.181 0.000	0 0 0 0 0 0 0 1	0 0 0 0 0 0 0 0	67.5 66.4 67.5 53.6 67.5 67.5 67.5 67.5	3

Dynamic fall Standing position Falling forward, Figure 3(d)	1.12 1.21 1.97 2.4	1.63 1.95 1.23 1.67	0.130 0.134 0.000 0.000	0.130 0.134 0.000 0.215	0 0 0 0	0 0 1 0	51.3 67.5 67.5 67.5	1

Dynamic fall Standing position Falling to the left, Figure 3(e)	1.33 2.06 2.73 2.71 2.72 2.72	1.42 1.97 1.83 1.83 1.81 1.81	0.281 0.391 0.407 0.317 0.328 0.340	0.187 0.391 0.407 0.317 0.328 0.340	0 0 0 0 0 0	0 0 0 0 0 0	43.5 67.5 67.5 67.5 67.5 67.5	3

Static fall Standing position Falling backward, Figure 4(b)	1.16 1.55 1.78	1.21 1.21 1.44	0.049 0.081 0.078	0.565 0.600 0.625	0 0 0	0 1 0	63.5 67.5 67.5	1

Static fall Standing position Falling backward, Figure 4(c)	1.30 1.85 1.88	1.40 1.90 1.49	0.222 0.335 0.152	0.177 0.401 0.656	0 0 0	0 0 0	48.5 62.7 67.5	3

Dynamic fall Standing position Falling to the right, Figure 4(d)	1.11 1.33 2.03 2.15	1.60 1.95 1.30 1.58	0.131 0.134 0 0	0.125 0.134 0 0.185	0 0 0 0	0 0 1 0	50.7 67.5 67.5 67.5	2

Dynamic fall Standing position Falling forward, Figure 4(e)	1.18 1.45 1.88	1.58 1.79 1.60	0.125 0.140 0.125	0.127 0.134 0.098	0 0 0	0 0 1	53.5 67.5 67.5	1

**Table 2:** Results of fall detection and fall pattern recognition from a “sitting” position in infrared and colour.

	Width to height ratio	Height change	Horizontal velocity	Vertical velocity	Fall direction	Position change	Fall	Fall pattern
Fall Sitting position Falling backward, Figure 3(f)	2.99 3.00 2.84 2.63 3.89 4.89	1.04 1.05 1.00 0.95 0.97 1.15	0.000 0.000 0.000 0.015 0.016 0.022	0.000 0.000 0.012 0.015 0.016 0.029	0 0 0 0 0 0	0 0 0 0 0 0	58.0 58.5 56.4 54.5 55.8 64.2	4

Fall Sitting position Falling to the right, Figure 3(g)	1.04 1.36 1.43 1.71 2.10 2.26 2.31	1.01 1.08 0.90 1.14 1.79 1.83 1.78	0.086 0.077 0.000 0.025 0.226 0.177 0.135	0.021 0.077 0.045 0.051 0.226 0.249 0.226	1 1 1 1 1 1 1	0 0 0 0 0 0 0	42.1 60.0 53.6 63.1 67.5 67.5 67.5	5

Fall Sitting position Falling backward, Figure 4(a)	1.85 2.00 2.15 2.16	1.11 1.05 1.25 1.43	0.027 0.021 0.058 0.154	0.011 0.010 0.047 0.221	0 0 0 0	0 0 0 0	58.05 57.57 55.41 55.33	4

**Table 3:** Result of fall detection and fall pattern recognition from a “lying” position and results of a false fall detection in infrared.

	Width to height ratio	Height change	Horizontal velocity	Vertical velocity	Fall direction	Position change	Fall	Fall pattern
Fall Lying position Falling backward, Figure 3(h)	3.03 3.02 3.56 4.14 4.15 4.22	1.21 1.12 1.12 1.27 0.98 1.05	0.000 0.000 0.816 1.014 0.000 0.000	0.182 0.317 0.633 0.713 0.013 0.032	1 0 0 0 1 1	1 1 1 1 1 1	38.6 39.5 67.5 67.5 53.0 58.0	7

False fall Kneeling, Figure 3(i)	2.10 1.93 1.95 1.97 2.04 2.27 1.92	2.16 2.02 1.00 1.01 1.04 0.78 0.68	1.141 1.117 0.602 0.731 0.731 0.423 0.507	0.439 0.465 0.200 0.285 0.303 0.141 0.282	0 0 0 0 0 1 1	0 0 0 0 0 0 0	32.5 35.9 33.7 22.4 0.0 0.0 0.0	
